# Eco‐evolutionary factors that influence its demographic oscillations in *Prochilodus costatus* (Actinopterygii: Characiformes) populations evidenced through a genetic spatial–temporal evaluation

**DOI:** 10.1111/eva.13544

**Published:** 2023-04-14

**Authors:** Sandra Ludwig, Juliana da Silva Martins Pimentel, Leonardo Cardoso Resende, Evanguedes Kalapothakis

**Affiliations:** ^1^ Departament of Genetics, Ecology and Evolution Federal University of Minas Gerais Belo Horizonte Brazil; ^2^ Faculdade Pitágoras Belo Horizonte Brazil

**Keywords:** *adegenet*, Bayesian Skyline Plot, evolution, historical demography, spawning waves

## Abstract

The human activity impact on wild animal populations is indicated by eco‐evolutionary and demographic processes, along with their survival and capacity to evolve; consequently, such data can contribute toward enhancing genetic‐based conservation programs. In this context, knowledge on the life‐history and the eco‐evolutionary processes is required to understand extant patterns of population structure in *Prochilodus costatus* a Neotropical migratory fish that has been threatened due to loss and fragmentation of its natural habitat since 1960s promoted by the expansion of hydroelectric power plant construction programs. This study evaluated the eco‐evolutionary parameters that cause oscillations in the demography and structure of *P. costatus* populations. An integrated approach was used, including temporal and spatial sampling, next‐generation sequencing of eight microsatellite loci, multivariate genetic analysis, and demographic life‐history reconstruction. The results provided evidence of the complex interplay of ecological–evolutionary and human‐interference events on the life history of this species in the upper basin. In particular, spawning wave behavior might have ecological triggers resulting in an overlapping of distinct genetic generations, and arising distinct migratory and nonmigratory genetic patterns living in the same area. An abrupt decrease in the effective population size of the *P. costatus* populations in the recent past (1960–80) was likely driven by environment fragmentation promoted by the construction of the Três Marias hydropower dam. The low allelic diversity that resulted from this event is still detected today; thus, active stocking programs are not effective at expanding the genetic diversity of this species in the river basin. Finally, this study highlights the importance of using mixed methods to understand spatial and temporal variation in genetic structure for effective mitigation and conservation programs for threatened species that are directly affected by human actions.

## 1 INTRODUCTION

Investigating past demographic processes that have shaped current patterns of population structure is fundamental for understanding the relationship between their evolutionary history, population size, and genetic diversity, especially in species that are susceptible to anthropogenic disturbance (Carroll et al., 2018; Lourenço, Álvarez, Wang, & Velo‐Antón, 2017). Therefore, measuring the genetic diversity of species and their capacity to maintain diversity over time is a central goal of conservation genetics (Flanagan, Forester, Latch, Aitken, & Hoban, 2018; Phillips et al., 2013).

Many conservation and fisheries stock studies have used biotelemetry (acoustic and radiotelemetry) as a monitoring method of the behavior of migratory fish species, such as distributions, migration patterns, reproductive cycle, and habitat uses (DeCelles & Zemeckis, 2014; Godinho, Silva, & Kynard, 2017; Magalhães‐Lopes, Alves, Peressin, & Pompeu, 2018, 2019; Magalhães‐Lopes, Pompeu, et al., 2018). Besides, the genetic methods have been also used to estimate the migration and gene flow (Almada et al. 2017; Castilho, Cunha, Faria, Velasco, & Robalo, 2017), improving our understanding about the demography and evolution life‐history studies of migratory fish populations (Mattingsdal et al., 2019; Pavlova et al., 2017). Especially in cases that there were habitat loss and fragmentation of riverbeds that influence not only the behavior but also the population dynamics (Pimentel & Ludwig, in press), thus, the genetics have been complementary implemented to ecological knowledge (Piorski et al., 2008; Savary et al., 2017).

The Neotropical migratory fish *Prochilodus costatus* (Valenciennes, 1,850) is a Brazilian species that is of commercial importance in the São Francisco River basin (Sato & Godinho, 2004). However, the hydropower dam of Três Marias was constructed in this region between 1954 and 1962 (Andrade, Prado, Loures, & Godinho,^2012^). Despite the social and economic benefits of hydropower dams, they often impact the genetic diversity of local fish populations (Baggio, Araujo, Ayllón, & Boeger, 2018; Carvalho‐Costa, Hatanaka, & Galetti, 2008) by hindering migration and altering gene flow dynamics, leading to population fragmentation (Horreo et al., 2011). Since there is no transposition system to reconnect the migratory fish populations from the downstream to upstream of the Três Marias dam, stocking programs have been developed as mitigating measure and implemented since 2003 using the *P. costatus,* attenuating such impacts in the São Francisco River basin (Agostinho, Pelicice, Gomes, & Júlio, 2010). Recently, Pimentel et al. (in press) genetically evaluated more than 1,000 individuals of *P. costatus* in the surrounding of the Três Marias dam, and detected similar genetic patterns from the downstream with the upstream populations and evidenced that the stocking program has connected and promoted artificially gene flow between the populations. However, it was demonstrated that the choice of reproductive matrices deserves greater attention to maintain/expand the genetic diversity of fish in the region.

The genetic structure of the population of *P. costatus* in the São Francisco River basin has been recently investigated (Pimentel et al., in press). However, the life history and the interactions and feedback between the environmental stochastic events and evolutionary mechanisms (herein threatened as eco‐evolutionary processes; i.e., Pelletier, Garant, & Hendry, 2009) that have shaped the current pattern population structure have not been clarified. According to Pimentel et al. (in press), the stocking program captured individuals from distinct downstream areas and breed them into the facilities without genetic criteria, with the argument that mixing between individuals from different regions would promote higher birth rates since they are not related. They also demonstrated that the stocking program maintained and/or homogenized the genetic pools in both regions but did not increase the genetic variability of the populations. Thus, to enhance conservation programs of migratory fish, more research must be done to improve the knowledge about the factors (i.e., environmental stochastic and evolutionary) that have shaped past and contemporary patterns of genetic diversity and structure of populations.

Several approaches have been improved to analyze environmental stochastic events and evolutionary mechanisms, that are based on coalescence reconstruction and tools from computational statistics, including moment matching (Kaut & Wallace, 2003), population decline and growth detection (Cornuet & Luikart, 1996), and likelihood approaches with varying effective population sizes (*Ne* of Wright, 1931) (Gilbert & Whitlock, 2015) based on contemporary and past *Ne* (Drummond, Rambaut, Shapiro, & Pybus, 2005; Waples, 1989; Waples & Yokota, 2007). These approaches have helped us to improve our knowledge about how evolutionary processes influence the life history of organisms (i.e., demographic events), in the wild (Hapeman, Latch, Rhodes, Swanson, & Kilpatrick, 2017; Perrier, Guyomard, Bagliniere, Nikolic, & Evanno, 2013; Pil et al., 2018). Likewise that have been recently affected by selection/anthropogenic pressures, such as rapid contemporary climate change (Crozier & Hutchings, 2014), habitat degradation and disconnection (Lourenço et al., 2017), (re)introduction of species (Hapeman et al., 2017), and/or stocking programs (Hansen, Fraser, Meier, & Mensberg, 2009).

This study conducted a wide‐ranging spatio‐temporal investigation to evaluate the demography of *P. costatus* populations encompassing contemporary and past events that could help us to explain its eco‐evolutionary history from upper São Francisco River basin. Moreover, Magalhães‐Lopes, Pompeu, et al. (2018) suggested the existence of migration wave behavior in *P. costatus*, along the same stretch of the São Francisco River, based on telemetry studies performed during the spawning/reproductive period. Thus, in order to assess whether the previously postulated migration wave behavior reflects in different spawning patterns that generate genetically distinct subpopulations of *P. costatus* in upper São Francisco River basin, we separated the samplings over a period of four hydrological years (2013/2014, 2014/2015, 2015/2016, and 2016/2017) in reproductive/migratory and nonreproductive/nonmigratory periods over four broad regions. To hit these questions, this study used an integrative approach, including the genotyping of the populations using microsatellite loci and the comparison of contemporary and past *Ne* oscillations through BSP coalescence reconstruction. Thus, these mixed approaches are expected to allow us to identify the main processes that have shaped the contemporary demography and genetic patterns of *P. costatus* populations that include biotic stress, fragmentation of the habitat, and human interference.

## 2 MATERIALS AND METHODS

### 2.1 Prochilodus costatus and the upper São Francisco River basin

*Prochilodus costatus* is a migratory fish that is endemic of the São Francisco River basin in Brazil that belongs to the Prochilodontidae family (Castro & Vari 2004). Wild females of *Prochilodus costatus* spawn, in total type, an average of 250,000 oocytes in the water to be fertilized externally, and development will occur as eggs float downstream (Sato et al., 2003). The sex ratio between *P. costatus* females and males is 1:1.5 (Arantes, Santos, Rizzo, Sato, & Bazzoli, 2011). Because *P. costatus* has a very high phylogenetic similarity to *P. lineatus* (Chagas et al. 2016; Melo et al. 2018), and as already reported by Castro & Vari (2004), species of the genus *Prochilodus* bear a very similar resemblance in morphology and behavior. According to Arantes et al. (2011), the mean total length is 35.5 ± 11.2 cm, the mean weight is 761.9 ± 692.0 cm *females,* and a sex ratio is estimated in 1:1.5 for *P. costatus* populations of São Francisco River basin. The highest longevity estimated time for individuals of *P. lineatus* (congener of *P. costatus*) in *L*_50_ and *L*_100_ of 1.6 years and 3.8 years, respectively, and this species presented an estimated 6 cohorts (Barbieri, Salles, & Cestarolli, 2018; Sato et al., 2003; Vicentin, Rocha, Rondon, dos Santos Costa, & Súarez, 2012).

Wild populations of *P. costatus,* as other species of Neotropical migratory fish populations, may exhibit homing behavior for breeding and feeding sites (Magalhães‐Lopes, Pompeu, et al., 2018). It is classified as iteroparous (i.e., the fish return to their living areas after the spawning season) (Braga‐Silva & Galetti, 2016; Sato & Godinho, 2004), and they tend to return to previously occupied sites during the reproductive cycle after spawning (Godinho & Kynard, 2006; Godinho et al., 2017; Hatanaka, Henrique‐Silva, & Galetti, 2006). Thus, the upstream migration is essential to complete its reproductive cycle (Sato et al., 2003). Upstream migrations (upstream of the Três Marias dam) are most often done in the main river channel (São Francisco River), but also include in minor frequency the tributary channels (Pará and Paraopeba rivers) (Magalhães‐Lopes, Pompeu, et al., 2018). *Prochilodus costatus* is dependent on the seasonal hydrological cycle and photoperiod which stimulate fish migration up the river during the spawning season, because is determinant to the onset of gonadal maturation and the first rains determine the onset of migration (Agostinho, Gomes, Veríssimo, & Okada, 2004; Magalhães‐Lopes, Alves, et al., 2018). The reproductive cycle is from October to January and may extend to March (Magalhães‐Lopes, Pompeu, et al., 2018; Sato et al. 1996) that presents two very marked frequency peaks in late October and early November with a difference of 10 to 20 days between them, generating different egg cohorts within the same breeding season (Magalhães‐Lopes, Alves, et al., 2018; Sato et al. 1996). Thus, such cycle repeats as long as the individual is alive (Braga‐Silva & Galetti, 2016; Harvey & Carolsfeld, 2003; Sato & Godinho, 2004).

**FIGURE 1:**
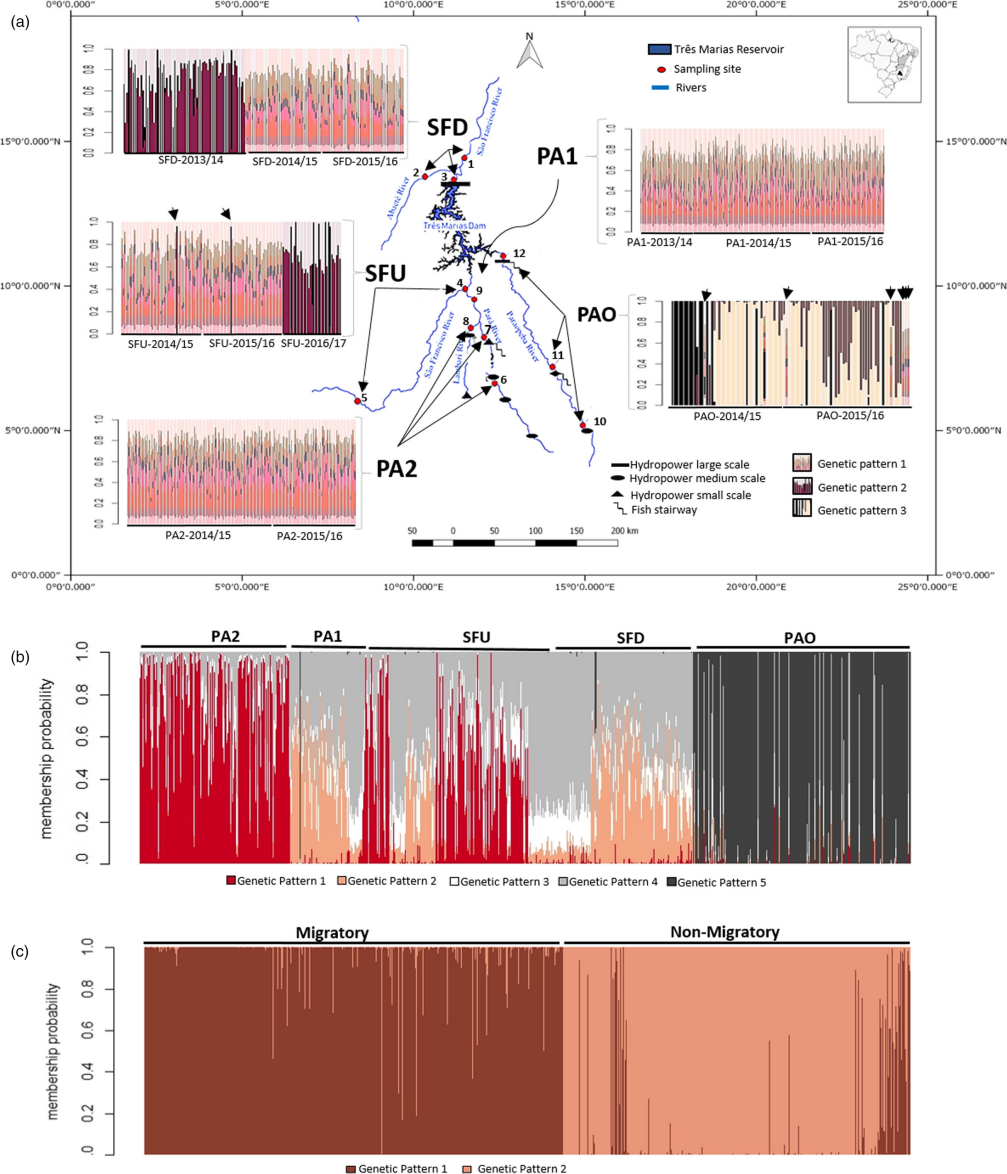
Distribution of the genetic patterns (= *k*‐clusters) of *Prochilodus costatus* populations from the upper São Francisco River basin, when comparing: (a) the hydrological years—SFD: 2013/2014, 2014/2015, 2015/2016; SFU: 2014/2015, 2015/2016, 2016/2017; PA2: 2013/2014, 2014/2015, 2015/2016; PA1: 2014/2015; 2015/2016; and PAO: 2014/2015, 2015/2016. (b) the areas—SFD: São Francisco River downstream; PA1: Pará River section 1; PA2: Pará River section 2; SFU: São Francisco River upstream; and PAO: Paraopeba River; and (c) the periods (migratory—PA2, SFU 2015/2016/2017, PAO, SFD 2016/2017; nonmigratory—SFD 2013/2014/2015/2016, SFU 2013/2014, PA1). The graphs show the membership probability of each individual (represented by one column) to be clustered in its respective genetic pattern (see legend of the colors). The arrows indicate individuals that presented divergent patterns. The sampling details are presented in Table 1

The upper portion of the São Francisco River is 630 km long and encompasses the river source (spring). Several hydropower dams are distributed in the upper São Francisco Riverbed, with the Três Marias being the largest (Godinho & Godinho 2003; Prado & Pompeu, 2014). The main tributary rivers contain the hydropower dams of Nova Dorneles, Cajuru, Gafanhoto, and Pitangui (in the Pará River) and Salto do Paraopeba, Igarapé, and Retiro Baixo (in the Paraopeba River) (Figure 1a) (Barroca et al. 2012; Godinho & Godinho, 2003).

### 2.2 DNA extraction and genotyping

In the upper São Francisco River basin, 1,027 specimens of *Prochilodus costatus* were sampled from 12 sites that were distributed into five major areas (SFD: São Francisco River downstream; PA1: Pará River section 1; PA2: Pará River section 2; SFU: São Francisco River upstream; and PAO: Paraopeba River) (Figure 1a) over a period of four hydrological years: 2013/2014, 2014/2015, 2015/2016, and 2016/2017 (Table 1). Samples were collected in accordance with the permanent collecting license of the Instituto Chico Mendes de Conservação da Biodiversidade (protocol number 57204‐1) and licensing from the Instituto Estadual de Florestas (protocol number 014.007 2017). The specimens were collected using a cast net and gillnet. A ~ 5 mm fragment of the caudal fin was cut before the fish were returned to the river. All caudal fragments were placed in separate 96‐well plates for subsequent genomic DNA extraction following the procedures of Pimentel et al. (2018).

**TABLE 1:** Sampling information of *Prochilodus costatus* and the genetic diversity indices from upper São Francisco River basin in Brazil

Areas^a^	River	Local^b^	Sites^a^	Hydrological year	Period	*N*	Ho	He	A	Na	G	*F*_is_
SFU	São Francisco River upstream	SRM	5	December−2016/2017	Migratory	22	0.43	0.6	9.24	4.88	0.62	0.232
			4,5	December −2015/2016	Migratory	14	0.31	0.49	6.76	3.65	0.59	0.285
		TM	4	September−2014/2015	Nonmigratory	39	0.3	0.58	10.02	4.5	0.58	0.372
			4	September −2013/2014	Nonmigratory	21	0.42	0.59	4.88	4.88	0.62	0.4
						96	**0.364**	**0.564**	**7.72**	**4.48**	**0.603**	**0.322**
PA2	Pará River	ITA	7	March−2014/2015	Migratory	32	0.33	0.42	3.23	3.18	0.5	0.214
			7	January−2013/2014	Migratory	45	0.27	0.45	3.27	2.1	0.52	0.303
		CPA	6	February−2014/2015	Migratory	31	0.33	0.42	3.92	4.25	0.54	0.247
			6	January−2013/2014	Migratory	15	0.35	0.54	3.29	4.3	0.57	0.319
		LAM	8	February−2014/2015	Migratory	23	0.33	0.43	3.74	4.32	0.45	0.191
			8	January −2013/2014	Migratory	25	0.36	0.59	3.84	5.2	0.61	0.346
						171	**0.33**	**0.47**	**3.55**	**3.89**	**0.53**	**0.3**
PA1	Pará River	MP	9	September−2015/2016	Nonmigratory	30	0.21	0.28	4.66	2.65	0.46	0.135
			9	September −2014/2015	Nonmigratory	49	0.31	0.56	12.73	5.88	0.58	0.375
			9	September −2013/2014	Nonmigratory	17	0.34	0.53	11.3	4.13	0.56	0.303
						96	**0.29**	**0.45**	**9.56**	**4.22**	**0.54**	**0.271**
PAO	Paraopeba River	SAL	10	December−2014/2015	Migratory	13	0.32	0.52	4.14	4.88	0.58	0.386
			10	December −2013/2014	Migratory	45	0.41	0.52	4.02	3.51	0.55	0.479
		IGA	11	December −2014/2015	Migratory	45	0.29	0.56	4.21	5.36	0.57	0.246
			11	December −2013/2014	Migratory	154	0.33	0.55	3.88	3.75	0.56	0.344
		RBA	12	December −2014/2015	Migratory	79	0.36	0.6	4.04	3.75	0.62	0.275
			12	December −2013/2014	Migratory	38	0.4	0.59	3.73	3.63	0.57	0.372
						374	**0.35**	**0.56**	**4**	**4.15**	**0.58**	**0.4**
SFD	São Francisco River downstream	TM	3	December −2016/2017	Migratory	34	0.43	0.6	3.38	2.67	0.48	0.285
			3	Sepember−2015/2016	Nonmigratory	135	0.36	0.52	8.63	5.75	0.52	0.219
			1,2	September−2014/2015	Nonmigratory	83	0.35	0.5	8.69	5.25	0.5	0.196
				April−2013/2014	Nonmigratory	18	0.39	0.56	7.48	6	0.57	0.248
						270	**0.38**	**0.54**	**7.05**	**4.92**	**0.52**	**0.237**

^a^ Areas and sites indicated in Figure 1.

^b^ Local of sampling (TM: Três Marias hydropower dam; SFU: São Francisco River upstream; SFD: São Francisco River downstream; SAL: Salto do Paraopeba; IGA: Igarapé; RBO: Retiro Baixo; MsP: Pará River mouth; LAM: Lambari River; CPA: Conceição do Pará; ITA: Itapecerica; SRM: São Roque de Minas). *N*: sampling number; Ho: observed heterozygosity; He: expected heterozygosity; A: allelic richness; Na: number of effective alleles; G: gene diversity; *F*_is_: fixation index. The numbers in bold represent mean values.

Genotyping was carried out using eight polymorphic microsatellite loci: *ProC10, ProC18, ProC22, ProC36, ProC37, ProC44, ProC48,* and *ProC49* (GenBank accession numbers: Proc10 MG456705; Proc18 MG456707; Proc22 MG456708; Proc36 MG456709; Proc37 MG456710; Proc44 MG456712; Proc48 MG456715; and Proc49 MG456716)*,* which protocol was developed and followed using Pimentel et al. (2018) instructions. The genotyping library was prepared using the 16S Metagenomic Sequencing Library Preparation^®^ Kit, with the addition of 5 µl DNA (4 nM) and 5 µl 0.2 M NaOH, followed by 5‐min incubation for DNA strand denaturation. Next, the genotyping libraries were diluted using Illumina buffer to obtain a final concentration of 10 pMol DNA in a final volume of 1 ml. Sequencing was performed with 600 µl of the final solution, using the MiSeq platform (Illumina^®^).

### 2.3 Data treatment with bioinformatics

For the detection and genotyping of microsatellites in the reads, the pipeline of Pimentel et al. (2018) was used and followed five steps: (1) trimming of the raw reads to remove the Illumina adapters and then filtered by the ratio of Phred‐scale probability of the genotype to sequencing depth (Phred = 30) and by length (>75 bp) using Prinseq‐Lite (Schmieder & Edwards, 2011); (2) alignment of the reads against the reference genome contigs of the *P. costatus* (all sequencing data are available at NCBI (NCBI Bioproject accession number: PRJNA548358)) using the high sensitivity prior in Bowtie 2 (Langmead & Salzberg, 2012), this step certifies that the reads are allocated within the target repeat region within the genome reference; (3) a local mapping to feasible the detected reads within the target repeat region with an average mapping efficiency of 96.3%, using the Realign Target Creator and Indel Realigner tools of the GATK package (McKenna et al., 2010); (4) identification of alleles per read, per microsatellite locus, and per an individual with a minimum depth x10 using the RepeatSeq tool (Highnam et al., 2012) (Supporting Information III); and (5) conversion of the multilocus genotype into the input.gen that is required by molecular and population genetics programs. Posteriorly, the proportion of null alleles of each locus using the EM algorithm (Dempster, Laird, & Rubin, 1977) of the results were checked using FreeNA (Chapuis & Estoup, 2006).

### 2.4 Genetic population analysis

To estimate the genetic diversity of *P. costatus* populations, we measured the observed (*Ho*) and the expected (*He*) heterozygosity, and to estimate the allelic richness (*A*), we performed a bootstrap through 999 replicates using the *diveRsity* package (Keenan, McGinnity, Cross, Crozier, & Prodöhl, 2013). Unbiased gene diversity (Nei, 1978) was performed in *poppr* package (Kamvar, Tab ima, & Grünwald, 2014). Both analyses were conducted in R software (R Development Core Team, 2008). Moreover, the conformance to Hardy–Weinberg (HW) expectations and linkage disequilibrium were checked using exact tests implemented in GenAlex v6.5 (Peakall & Smouse, 2006), and using FreeNA (Chapuis & Estoup, 2006) the proportion of null alleles of each locus through the EM algorithm (Dempster et al. 1977).

To evaluate the genetic structure of the *P. costatus* population, we separate the dataset in three distinct combinations: (1) by hydrological years (2013/2014; 2014/2015; 2015/2016; and 2016/2017); (2) by areas (SFU, SFD, PA2, PA1, and PAO); and (3) by migratory/reproductive and nonmigratory/nonreproductive periods. Such combinations were independently analyzed by three distinct methods and softwares: AMOVA, pairwise *ϴ*st (Weir & Cockerham, 1984), and discriminant analysis of principal components (DAPC) (Jombart, Devillard, & Balloux, 2010). The AMOVA was performed in GenAlex, the pairwise *ϴ*st was performed in GENETIX4.05 (Belkhir, Borsa, Chikhi, Raufaste, & Bonhomme, 2004), and DAPC was performed through *adegenet* R package (Jombart, 2008). To perform the DAPC, in a first step, the dataset is evaluated through the *find.clusters* function that transforms the genotypes into uncorrelated components using principal component analysis (PCA) and then run the *k*‐means algorithm to detect the range of possible *k*‐clusters (without any a priori information of population); thus, the individuals are assigned into those *k*‐clusters using Bayesian information criterion (BIC). In a second step, a discriminant analysis (DA) is performed through *dapc* function using the number of retained PCs in order to maximize the variation of the obtained *k*‐clusters. Then, a membership probability of each individual is performed to be grouped into their respective *k*‐clusters through *compoplot* function that results in an assignment a probability (ranging from 0% to 100%) of an individual to belong into their *k*‐cluster (obtained in the previous step of the analysis).

Further, to exclude potential population structure due to relatedness, we estimated in COANCESTRY v1.0.0 (Wang, 2011) using the method of Lynch and Ritland (1999), different relatedness classes per population considering *r* ≤ 0.25 as unrelated, *r* between 0.26–0.5 as half‐siblings, and *r* ≥ 0.5 as full siblings as suggested by Jones, Small, Paczolt, and Ratterman (2010), and significance was tested via 1,000 bootstrap replicates.

### 2.5 Demographic analysis

The contemporary effective population size (*N*e) of the *P. costatus* populations was estimated using the linkage disequilibrium (LD) method (Waples, 1989) and compared with the temporal estimator method (Tp) (Waples & England, 2011). These methods were chosen because LD assumes that random departures from linkage equilibrium of unlinked loci are inversely proportional to *N*e and takes into account the sample size (Waples & Do, 2008), while the Tp calculates a standardized variance in the temporal changes of allele's frequencies comparing temporal‐spaced samples that attributing also the lowest allele frequency criteria, based on Pollak (1983) which let us set as 0.05 (Wang, Santiago, & Caballero, 2016). Both methods were performed through the software NeESTIMATOR 2.0 (Do et al., 2014) because of its superior performance in ideal scenarios (Gilbert & Whitlock, 2015), when migration rates are moderate and the sample size is not fixed, the sex is uneven, and the population is subdivided, which fits with our study system. Confidence intervals (CIs) were obtained from the Jackknife option.

To assess whether the population showed the signature of a recent demographic event (i.e., recent expansion or a population bottleneck), the software BOTTLENECK v1.2.02 (Piry, Luikart, & Cornuet, 1999) was used because it calculates the expected distribution of allele frequencies and the significant number of loci with heterozygosity excess. Two different mutation models were tested: infinite allele model (IAM; Maruyama & Fuerst, 1985) and strict stepwise mutation model (SMM; Cornuet & Luikart, 1996). BOTTLENECK uses the contemporary *Ne* to test for the presence of heterozygosity excess, as would be expected following a bottleneck event. According to Piry et al. (1999), BOTTLENECK software can detect reductions within the past 2*Ne*–4*Ne* using microsatellites loci. Thus, populations that have suffered a bottleneck event are expected to show excess heterozygosity in comparison with the number expected under mutation‐drift equilibrium, in addition to showing reduced abundance of low frequency allele classes (“mode‐shifted”). This phenomenon represents the loss of rare alleles and could be assessed by looking at the overall distribution of allele frequency classes (Cornuet & Luikart, 1996). The significance of excess heterozygosity was assessed using a one‐tailed Wilcoxon sign‐rank test, as recommended for fewer than 20 loci (Luikart, 1997), and probability (*P*) for 1,000 simulations.

Past demographic events were inferred based on variation in *Ne* over the past generations of *P. costatus* populations from the upper São Francisco River basin using the coalescent approach, which was based on BSP implemented in the VarEff R package (Nikolic & Chevalet, 2014). This parameter estimates the oscillations of *Ne* using microsatellite markers. The estimation is performed on simulated demographic histories modeled by steps of constant size for which the posterior probabilities are derived using an approximate likelihood Markov Chain Monte Carlo (MCMC) approach. This analysis simulated past demography using microsatellite data with a coalescent approach, to estimate changes in recent and ancestral *Ne*. This approach relies on motif distance frequencies between alleles to estimate variation in *N*e over time (global θ = 4*Ne*µ; µ = mutation rate) (Nikolic & Chevalet, 2014). Demographic changes were modeled with MISAT software (Nielsen, 1997) using a two‐phase mutation model with µ = 5.0 × 10^–6^ (estimated mutation rate) and 10% of the mutations greater than a single step. Then, we set four independent MCMC runs simulating for 10 million generations sampled at every 1,000 trees and 25% burn‐in. The sexual generation time for *P. costatus* was set to four years, as indicated by Santos and Barbieri (2015), and we transformed the generation times in time in the past as a pattern. The number of generations ranged from 100 to 1,000 since the origin of each population. For each population, models were run with three separate prior *N*e (minimum, intermediate, and maximum values) through NBAR function that attributes three global θ as means of effective size being: θ0 of 2.3384811908619 as *Ne* minimum, θ1 of 10.4918537554347 as *N*e intermediate, and θ2 of 12.5949077154245 as *N*e maximum. In the last 500 generations, changes to *Ne* were assessed. Inferences were made about recent past expansions and declines based on posterior distributions and their attributes (mean, median, mode, and harmonic values), as suggested by Nikolic and Chevalet (2014a, 2014b). The results were displayed graphically, using the *ggplot2* R package (Wickham, 2011), for the last 100 years and with a 95% confidence interval (CI).

## 3 RESULTS

### 3.1 Genetic diversity and structure

During the four hydrological years of sampling in the upper São Francisco River basin (including migratory and nonmigratory periods), 1,027 specimens of *P. costatus* were collected from 12 sites across the three main rivers (São Francisco, Pará, and Paraopeba) (Table 1; Figure 1). Of these specimens, 1,017 were successfully genotyped, and their genetic diversity was investigated using eight microsatellite loci. Comparing the hydrological years, the lowest *A* was observed for PA2‐ITA (2014/2015), and highest *A* was observed for PA1‐MP (2014/2015) (Table 1). Comparing the areas, the lowest *A* mean was observed in PA2 and the highest in PA1, both are located in Pará River (Table 1). Comparing the nonmigratory periods, PA1‐2015/2016 showed the lowest *A* = 4.66, while PA1‐2014/2015 showed the highest *A* = 12.73 (Table 1). In comparison, for the migratory periods, the lowest mean genetic values were observed in PA2 2014/2015 and the highest in SFU‐SRM 2016/2017. Furthermore, we detected high allelic and gene diversity, but the *Na* per area was on average low (Table 1). A slight decline in the frequency of private alleles was also observed, which declined to zero in 2015/2016 and 2016/2017 (Supporting Information I). Moreover, the loci used in this study were not in conformance to Hardy–Weinberg (HW) expectations, and the proportion of null alleles were not significant < 0.09 (Supporting Information IV).

The DAPC resulted in distinct genetic patterns depending on the combination of the dataset (Figure 1), attributing three genetic patterns (*k* = 3) comparing the hydrological years (Figure 1a), five genetic patterns (*k* = 5) comparing the areas (Figure 1b), and two genetic patterns (*k* = 2) comparing the periods (Figure 1c). In Figure 1a, the most frequent genetic pattern (1) was observed in almost all the populations that were sampled during the years of 2014/2015 and 2015/2016. A second genetic pattern (2) was detected in SFD‐2013/2014 and SFU‐2016/2017, and was also present in one individual of SFU‐2014/2015 and one individual of SFU‐2015/2016 (indicated by the arrows in Figure 1a). In Figure 1b, the most frequent genetic pattern (1) was observed in almost all the areas except in PAO, which presented a unique genetic pattern (5), with seven individuals showing the genetic pattern 3. In Figure 1c, there are two markedly distinct genetic patterns, with the detection of some individuals from the migratory period with a genetic pattern of the nonmigratory period, and vice versa. For a double check of the DAPC results about the distinct genetic pattern observed for migratory and nonmigratory, we performed a factorial correspondence analysis in GENETIX (see Supporting Information II).

These results were corroborated by AMOVA, which demonstrated significant genetic structuring among the sampling years (*ϴ*st = 0.101, *p* = .01), among individuals of sampling areas (*ϴ*it = 0.586, *p* = .01), between the migratory and nonmigratory periods (*ϴ*st = 0.065, *p* = .01), and among individuals between the migratory and nonmigratory periods (*ϴ*it = 0.621, *p* = .01) (Table 2). The results θ_ST_ for SFD sample that was collected in 2013/2014 showed a significant difference when compared with all the other samples, except SFU‐2016/2017. Between 2014/2015 and 2015/2016, no significant difference was observed among any of the populations. The only exception was the SFD‐2014/2015 and SFU‐2015/2016 and the PAO populations, which exhibited significant differences in comparison with all other populations, independent of the sampling year (diagonal below Table 3). Pairwise comparisons among areas showed genetic structuring among the areas, except for PA1 and PA2 (diagonal above in Table 3).

**TABLE 2:** AMOVA comparing hydrological years, areas, and migratory and nonmigratory periods analyzed for *Prochilodus costatus* populations in the upper São Francisco River basin in Brazil

	*df*	MS	Est. Var.	%	*F* a
Hydrological years					
Among years	13	41.021	0.267	10%	**0.101**
Among individuals	1,001	3.797	1.419	54%	0.597
Within individuals	1,015	0.959	0.959	36%	0.637
Areas					
Among regions	15	29.717	0.299	12%	0.117
Among individuals	714	3.567	1.318	52%	**0.586**
Within individuals	730	0.932	0.932	37%	0.634
Migratory × nonmigratory					
Among periods	1	182.509	0.176	7%	**0.065**
Among individuals	1,013	4.098	1.570	58%	**0.621**
Within individuals	1,015	0.959	0.959	35%	0.645

^a^ *p*‐Values < 0.01 are highlighted in bold.

**TABLE 3:**
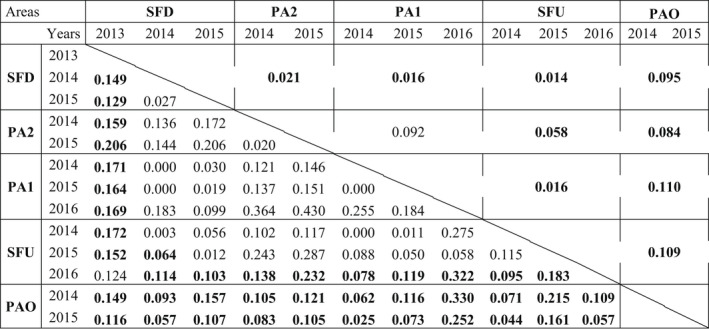
Pairwise *θ*st comparing the populations of *Prochilodus costatus* of upper São Francisco River basin, between years (diagonal below) and areas (diagonal above)

Note

The bold numbers indicate the significative *p‐*value = .01.

The relatedness analysis was significantly higher between pair of individuals sampled between years (*r* = 0.391, variance ± 0.165) than between areas (*r* = 0.276, variance ± 0.034) and between populations (*r* = 0.164, variance ± 0.082), being 51% of the individuals are full siblings, 36% are half‐siblings, and 13% are unrelated.

### 3.2 Demographic results

Comparing the areas, the PAO presented the highest (mean *Ne* = 215.4) and PA1 presented the lowest (mean *Ne* = 13.8) for LDNe, and for TpNe the PAO also presented the highest (mean *Ne* = 265.1) and PA1 the lowest (mean *Ne* = 10.1) that were obtained through NeESTIMATOR (Table 4). A noticeably low *Ne* was detected in SFD (LDNe = 2.94 and TpNe = 1.6, *N* = 34), which represent the site in the original riverbed of the São Francisco River basin, in the hydrological year 2016/2017. When the mean *Ne* for LDNe of all the populations was compared among the hydrological years, it increased from 42.06 (*n* = 378) in 2013/2014 to 61.67 (*n* = 394) in 2014/2015, followed by an abrupt decrease to 25.03 (*n* = 179) in 2015/2016 and 9.97 (*n* = 56) in 2016/2017 (Supporting Information I). In contrast to the LDNe, the TPNe resulted in low values for *Ne* although the CI (95%) reached more range of variation (Table 4).

**TABLE 4:** Demographic results for *Prochilodus costatus by years and* populations from upper São Francisco River basin in Brazil based on LDNe and TpNe estimative and its CIs (95%) obtained through NeEstimator; and Wilcoxon sign‐rank test and its probability to IAM and SMM obtained through Bottleneck

Areas	Local	Hydrological years	NeEstimator				Bottleneck	
			LDNe		TpNe		Wilcoxon	
			*Ne*	CI (95%)	*Ne*	CI (95%)	IAM (*p*‐value)^a^	SMM (*p*‐value)^a^
Comparisons by areas and years								
SFU	SRM	December−2016/2017	17	14.9–20.1	15	4.8–∞	**.309**	.127
		December −2015/2016	8.7	6.6–9.3			**.375**	.449
	TM	September−2014/2015	17.4	12.1–54.5	28.1	17.1–∞	**.358**	.524
		September −2013/2014	13.4	12.7–29.5			**.259**	.069
PA2	ITA	March−2014/2015	18.5	7.9–24.1	7.4	9.2–∞	**.298**	.624
		January−2013/2014	41.4	25.6–243.1			**.221**	.261
	CPA	February−2014/2015	17.5	14.6–36.6	14.6	26–∞	**.239**	**.087**
		January−2013/2014	11.5	9.2–12.3			**.242**	.375
	LAM	February−2014/2015	16.8	10.6–27.8	19.6	8.1–297.6	**.375**	.307
		January−2013/2014	24.5	17.4–45.9			**.037**	.263
PA1	MP	September −2015/2016	8.2	5.6–12.4	10.1	3.3–26.8	.255	**.066**
		September −2014/2015	20.1	19.6–28.8			**.625**	**.251**
		September −2013/2014	13.1	10.1–13.4			**.467**	.375
PAO	SAL	December −2014/2015	140.2	108.0–∞	96.4	79.3–371.9	.308	.195
		December −2013/2014	75.2	66.5–111.8			**.217**	.404
	IGA	December −2014/2015	192.1	150.3–∞	164.2	199.5–∞	**.506**	.291
		December −2013/2014	52.6	66.5–131.5			**.446**	.004
	RBA	December −2014/2015	140.2	102.0–∞	65.1	79.3–269.7	**.049**	.235
		December −2013/2014	165.2	116.5–214.7			**.261**	.476
SFD	TM	December −2016/2017	2.94	1.8–9.6	1.6	2.0–∞	.133	**.127**
		September −2015/2016	58.2	35.3–131.9			**.417**	.449
		September −2014/2015	44.2	42.1–∞	16.8	4.2–66.1	**.563**	.136
		April−2013/2014	49.1	19.3–∞			**.625**	.069
Comparisons by populations^b^								
Pop1	SFD	April−2013/2014	624.0	66.0–∞	Pop1 × Pop2	1.1–22.0	**.625**	.069
	SFD	December −2016/2017					.133	**.127**
	SFU	December −2016/2017			4.7		**.309**	.127
Pop2	SFD	September −2014/2015	540.0	113.4–∞	Pop 2 × Pop3	3.6–7.4	**.563**	.136
	SFD	September −2015/2016					**.417**	.449
	PA1	All years			5.2		**.449**	.231
	PA2	All years					**.235**	.319
	SFU	September −2014/2015					**.358**	.524
	SFU	December −2015/2016					**.375**	.449
Pop3	PAO	All years	358.6	115.3–∞	Pop 3 × Pop 1	1.3–3.5	**.297**	.267
					2.2			

^a^ Numbers in bold demonstrate significative values for *p* ≤ .05.

^b^ Stipulated by DAPC results showed in Figure 1a.

BOTTLENECK results were similar independent of the comparisons among hydrological years or areas or periods, and independent of the mutation model tested (Table 4). A recent population expansion was detected when the areas were compared based on the detection of an excess of heterozygosity detected for all sites, except PA2‐2015/2016 and PA1‐2013/2014. However, a recent bottleneck event was observed in SFD‐2013/2014 (*p* ≤ .05) and PA2‐LAM‐2014/2015 (*p* ≤ .02) when the hydrological years were grouped, and in SFU (*p* ≤ .01) and PA2 (*p* ≤ .02) when analyses were performed with respect to area (Table 4).

**FIGURE 2:**
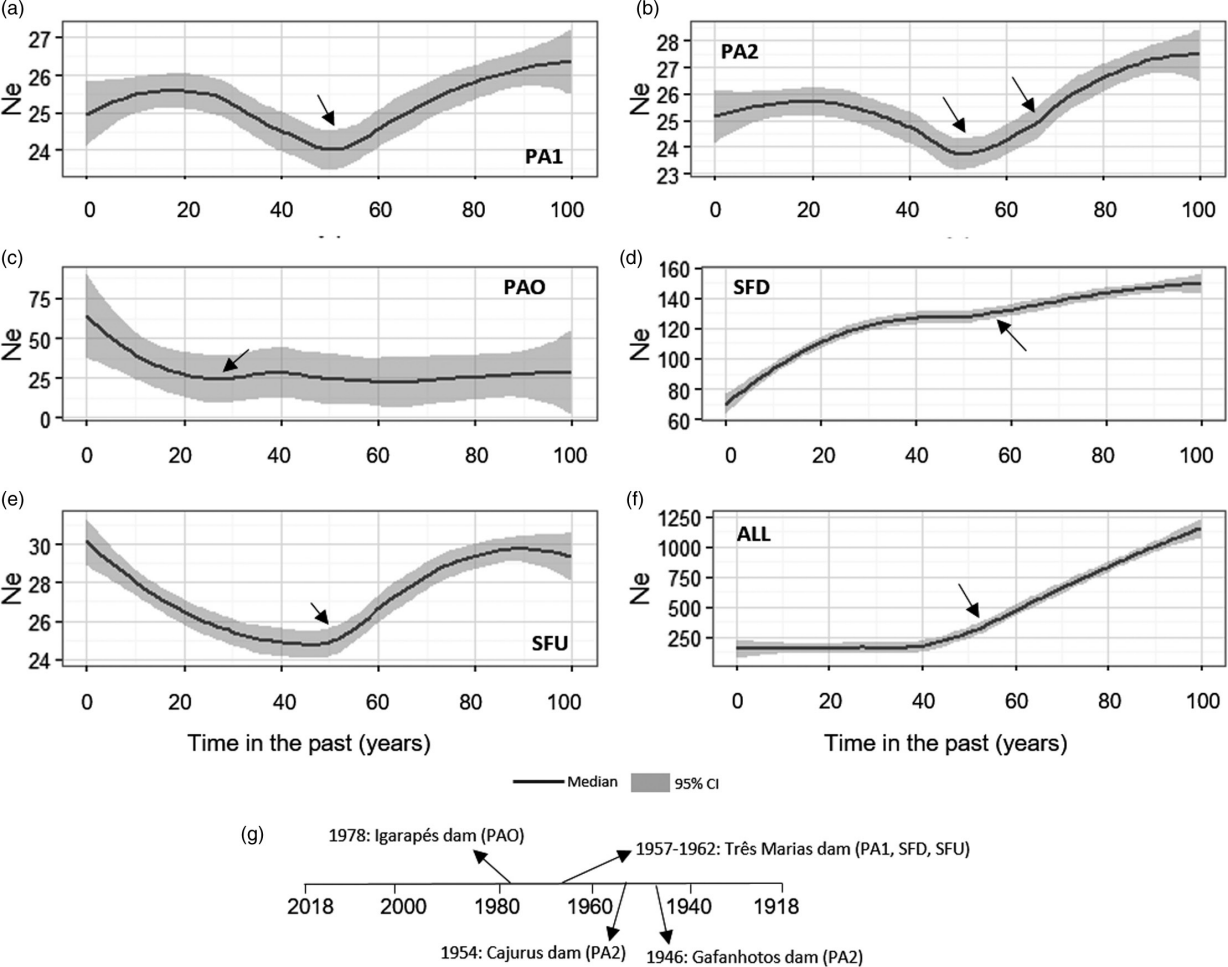
Past demographic oscillations of *Prochilodus costatus* effective population size (*Ne*) assessed by the Bayesian *Skyline Plot* (using VarEff R package) in the last 100 years (500 generations). The arrows indicate the bottleneck events. The estimated past *Ne* is shown for *P. costatus* populations in (a) PA1, (b) PA2, (c) PAO, (d) SFD, (e) and SFU, and (f) shows the estimated *Ne* to all populations combined. Note that the y‐axis (*Ne*) has a different scale in each plot

The historical demographic results showed bottleneck events in the recent past for the *P. costatus* populations (Figure 2). The population size remained constant in all areas between 100 and 80 years ago. However, bottleneck events were detected in the last 70 years (indicated by the arrows in Figure 2a–f). One bottleneck event was detected in PA1 between 40 and 30 years ago (Figure 2a), and in SFD between 40 and 20 years ago (Figure 2d). Two bottleneck events were detected in PA2 between 70 and 60 years ago and between 30 and 20 years ago (Figure 2b), and in PAO between 60 and 50 years ago and between 40 and 30 years ago (Figure 2c). The PAO and SFD populations exhibited population expansion in the last 30 years (Figure 2c; 2D). Of note, PA1 was markedly impacted by the bottleneck, with a consequent reduction in *Ne* to less than 10 individuals (Figure 2a). Overall, the *P. costatus* populations from the upper São Francisco River basin were impacted by extreme bottleneck events in the recent past, between 1960 and 1980 (Figure 2f).

## 4 DISCUSSION

### 4.1 Low genetic diversity

This study delineated the genetic structure of *P. costatus* populations across the main rivers of the upper São Francisco River basin between migratory and nonmigratory periods over four hydrological years (2013/2014 to 2016/2017). The data demonstrated intra‐annual stability between 2013/2014 and 2016/2017 based on DAPC and AMOVA. However, despite similarity among the different methods used to assess temporal and spatial stability at sampling sites, intra‐year comparisons produced fewer significant differences compared to across years. Comparing the average *He* (He = 0.54) detected herein for the *P. costatus* populations with those founded by Carvalho‐Costa et al. (2008) (He = 0.80) during the hydrological year 2003/2004 for the same stretch of the basin, the decreased inter‐population heterozygous may be consequence of the selection pressures that might be acting on the frequency of some loci, and, possibly, affecting intra‐year allelic and heterozygosis frequencies. Nevertheless, another possible explanation could be considered a consequence of genetic drift that decreased the allele's frequencies over the sampling years.

The high *Ne*, *He,* and *A* recorded for PAO reflected the genetic values expected for all populations of the São Francisco basin, since according to Lowe, Kovach, and Allendorf (2017) the high *Ne* is directly correlated with the high *He*, which reflects the predominance of heterozygotes in the population and indicates high intrapopulation genetic diversity. In this way, PAO exhibited a unique and more diversified genetic pattern (genetic pattern 3 in Fg. 1A and genetic pattern 5 in Fi. 1B) that was obtained through DAPC that may indicate that even with the construction of the Retiro Baixo dam in 2011 (Cachapuz, 2006), there is no subdivision in this population when compared to all other evaluated populations for the area of São Francisco River. Thus, it is likely that the unique genetic pattern and the distinct genetic diversity indexes observed in the PAO reflect the recent implementation of the dam. In addition, small‐scale stocking programs were implemented in the PAO since 2002 and until 2013, and fishing has been banned in the river since 2004 by state law (Decree nº 43.713, January 14, 2004); thus, of note, the PAO had higher population levels than the other sampled areas (Calazans, Pinto, Costa, Perini, & Oliveira, 2018; De Vilhena, Abreu, Lobato, Schayer, & Nonato, 2008; Veado et al., 2006).

Stocking programs have been successfully applied as a mitigation measure to connect migratory fish populations in the São Francisco River basin (Queiroz et al., 2016; Savary et al., 2017). However, the current study showed that *Ne* and *He* remain low in all of the populations in this stocking area, despite restocking programs being implemented for *P. costatus* in SFD, SFU, PA1, and PA2 between 2002 and 2013 (technical data provided by the Companhia de Desenvolvimento dos Vales do São Francisco e do Parnaíba—CODEVASF). Several studies of repopulation with native salmonids in various parts of the world have shown that stocking has little impact on the genetic makeups of populations (e.g., Eldridge & Killebrew, 2008; Gow, Tamkee, Heggenes, Wilson, & Taylor, 2011; Small, Currens, Johnson, Frye, & Von Bargen,^2009^; Stelkens, Schmid, Selz, & Seehausen, 2009). In contrast, other works have suggested that intense fish stocking can lead to increased genetic diversity (e.g., Ferreira et al. 2017; Marie, Bernatchez, & Garant, 2010; Valiquette, Perrier, Thibault, & Bernatchez,^2014^) and the introduction of genes, thereby affecting the genetic rescue of endangered populations by alleviating inbreeding depression and boosting fitness (Ingvarsson, 2005; Tallmon, Luikart, & Waples,^2005^). The *Ne* values recorded in this study were noticeably low, with *Ne* representing the most refined estimate of the effectiveness of stocking programs toward increasing the genetic diversity of migratory fish populations. Thus, our data suggest that conservation programs involving stocking strategies could benefit from the genetic monitoring of *Ne*, allelic frequencies, and *He* of the reproductive matrices and their respective offspring over generations.

### 4.2 Spawning waves and ecological triggers

Spawning waves have been detected in migratory fish for decades (Hede Jørgensen, Hansen, & Loeschcke, 2005; Lambert, 1987; Wright & Trippel, 2009); however, only a few studies have explored the genetic evidences of such behavior (Hede Jørgensen et al., 2005; McPherson, Stephenson, & Taggart, 2003). In the start of the spawning, migratory fish start upstream migration, which promotes sexual maturation and initiates the reproduction period (Wright & Trippel, 2009). Iteroparous migratory fish, such as *P. costatus* (Sato & Godinho, 2004), return to their living area after the spawning season (Braga‐Silva & Galetti, 2016). Thus, the living area of *P. costatus* might contain an overlapping of distinct generations or genetic subpopulations. McPherson et al. (2003) also documented such genetically distinct sympatric subpopulations in the Atlantic herring *Clupea harengus* Linnaeus, 1761, with this phenomenon being attributed to spawning waves. Our relatedness results showed that the evaluated individuals are in majority full siblings between years and areas, demonstrating us that there is a temporal and regional structure and that have been influenced by the genetic and demography of *P. costatus* in the upper São Francisco River basin. Furthermore, the detection of migrants with distinct genetic patterns indicates to us the presence of such subpopulations, which during the spawning seasons, exhibit distinct tempo‐regional genetic patterns to their progenies. Following migration and reproduction, heterogeneous larval pools with distinct allelic frequencies might be generated through the mixing of different living areas, similar to that suggested for other marine species (i.e., Selkoe, Gaines, Caselle, & Warner, 2006). The results obtained for migratory (SFU‐2016/2017, PA2‐2014/2015, and PAO‐2014/2015) and nonmigratory (SFU‐2013/2014/2015/2016, SFD‐2014/2015/2016, and PA1‐2014/2015/2016) periods in this study reinforce these findings, with the formation of markedly distinct genetic patterns. Thus, this study evidenced that there are distinct genetic patterns in *P. costatus* populations separated by migratory/reproductive and nonmigratory/nonreproductive periods—based on AMOVA, DAPC, and GENETIX (Supporting Information II)—which lead us to understand that they may represent sympatric subpopulations living in upper São Francisco River basin, suggesting that their populations exhibit a spawning wave behavior.

Spawning waves are directly stimulated by ecological triggers for migratory fish to reproduce and enhance offspring survival during the optimal season (Wright & Trippel, 2009). In general, migration is initiated when there is a combination of high organic matter and rainfall, which increases water turbidity and decreases predation risk (Magalhães‐Lopes, Alves, et al., 2018; Wright & Trippel, 2009). *Prochilodus costatus* populations migrate during the spring rainy season, when there is high river discharge (Alves, Vieira, Magalhães, & Brito, 2007; Magalhães‐Lopes, Alves, et al., 2018; Parkinson, Philippart, & Baras, 1999). In contrast, for *P. costatus* populations, nonideal environmental conditions, such as the narrowing of the riverbed, decrease, or even interrupt, the timing of migration and reproduction (Arantes et al., 2011; Sato & Godinho, 2004). The *P. costatus* population of the Três Marias reservoir was impacted by extended drought seasons during the hydrological years of 2001/2002 and 2002/2003, as well as a marked decrease in river volume between 2011/2012 and 2014/2015, when the river reached the lowest level historically (Abreu & Maillard, 2016). The narrowing of the riverbed during this period might explain the abrupt change to the genetic pattern observed in 2013/2014 to 2014/2015 and the temporal homogenization of populations during 2014/2015 and 2015/2016. The reduced rate of fish migration during periods of riverbed narrowing might also explain the occurrence of migratory genetic patterns during the nonmigratory periods and vice versa. Interestingly, the same genetic pattern observed in SFD‐2013/2014 was detected, again, in 2016/2017, coinciding with a considerable increase in river height after 2015/2016 (Portal *Hidroweb*, Agência Nacional das Águas). Thus, genetic pattern 2 might be the original pattern of the population. In comparison, genetic pattern 1 (observed throughout 2014/2015 and 2015/2016) was a consequence of the long drought period in the basin. However, only one site was sampled in 2016/2017, with additional sampling being required in the future to establish whether this hypothesis holds.

### 4.3 Demographic oscillations in the life history of *P. costatus*

Estimating contemporary and past *Ne* is important for suggests or infers loss diversity (in this case microsatellites) since it allows access to the decreasing of the *Ne*, the genetic variation levels, and its impact on wild population fitness under human interference (Palstra & Ruzzante, 2008). Nevertheless, the evolutionary implications of oscillations in *P. costatus* population demographics have not been explored by conservation studies (Pelicice et al., 2017; Piorski et al., 2008). The current study showed that the contemporary *Ne* of *P. costatus* populations in the São Francisco River basin has been oscillating in the last years (Supporting information I).

In comparison, past *Ne* reflects historical demographic oscillations, which also impact the allelic frequencies over time and are directly influenced by eco‐evolutionary interactions and population dynamics (Lowe et al., 2017). The current study provided evidence for an abrupt decrease in *Ne* in all *P. costatus* populations in our study region, with past bottleneck events occurring between 70 and 30 years ago. These bottleneck events were concomitant with the construction of several hydropower dams since 1946 in the upper São Francisco River basin (Cachapuz, 2006). In the original riverbed of the São Francisco River, the Três Marias dam was constructed between 1957 and 1962 (Cachapuz, 2006), and the river margins were flooded reaching PA1, SFD, and SFU areas (Sampaio & López, 2003). These actions impacted the *P. costatus* populations, as indicated by an extreme bottleneck event between 60 and 40 years ago in these areas (Figure 2a, d, e, g). Moreover, two bottleneck events were detected in PA2 between 70 and 50 years ago (Figure 2b), which were concomitant with the construction of the hydropower dams of Gafanhoto in 1946 and Cajuru in 1959 (Cachapuz, 2006) (Figure 2g). In PAO, the bottleneck event detected between 40 and 30 years ago (Figure 2c) was concomitant with the construction of the Igarapé thermal plant dam in 1978 (Alves et al., 2007) (Figure 2g). Thus, migration and connectivity between the populations downstream and upstream of the dams were blocked following the fragmentation of the original riverbed (Sato & Godinho, 2004). The transformation of the environment surrounding the dam severely impacted the *P. costatus* populations, with the genetic consequences still being perceived today. Therefore, the construction of the dams impacted the regulation of the river, which, in turn, impacted the migratory behavior of the *P. costatus* populations, and, possibly, other migratory species that spawn in the floodplains (Pompeu & Godinho, 2006). Although the *Ne* slightly recovered 30 years after the bottleneck events, the original equilibrium has yet to be reached. In conclusion, *P. costatus* populations are having difficulty overcoming the evolutionary pressure caused by the dams, with a longer period being required to reestablish the original genetic patterns.

## 5 CONCLUSIONS

This study highlighted the complex interplay of ecological and evolutionary events in shaping the life history of *P. costatus* populations in the São Francisco River basin, Brazil. *Prochilodus costatus* populations were subjected to an abrupt bottleneck event in the recent past (1960–1980), likely due to the environment fragmentation promoted by the construction of hydropower dams. Although stocking programs have been implemented to help address the impacts of dam construction in the area, these programs remain ineffective at expanding the level of genetic diversity, as low allelic diversity intrapopulation levels continue to be detected. Our results show that *P. costatus* populations are still subject to inbreeding depression, due to environmental changes caused by anthropogenic actions, raising the question of how long these populations will persist. This integrative study also emphasized the importance of using long‐term molecular methodologies to evaluate the eco‐evolutionary consequences of anthropic interference shaping the survival of wild organisms. This approach could be used to enhance mitigation programs and guide conservation decision‐making.

## CONFLICT OF INTEREST

None declared.

## Supporting information

Supporting Information

## Data Availability

Data underlying this study are available in Dryad: https://datadryad.org/stash/share/xovKEPenZcKgUgAAL4aLtsIRdR_4KphvAvYlCkG‐i_c.
