# Supplementary material for: Eco‐evolutionary factors that influence its demographic oscillations in Prochilodus costatus (Actinopterygii: Characiformes) populations evidenced through a genetic spatial–temporal evaluation
Source: Evol Appl. 2023 Apr 14;16(4):895–910. doi: 10.1111/eva.13544 (PMC10130561; doi:10.1111/eva.13544)
Supplement: Supplementary file 1 — Supporting Information [file EVA-16-895-s001.pdf]

Supporting Information

I. Genetic indexes observed for *Prochilodus costatus* populations sampled across the hydrological years analyzed in this study. NA: number of alleles; Eff NA: number of effective alleles; I Shannon's: Shannon's index; PA: private alleles; He: expected heterozygosity; and Ne: effective population size.

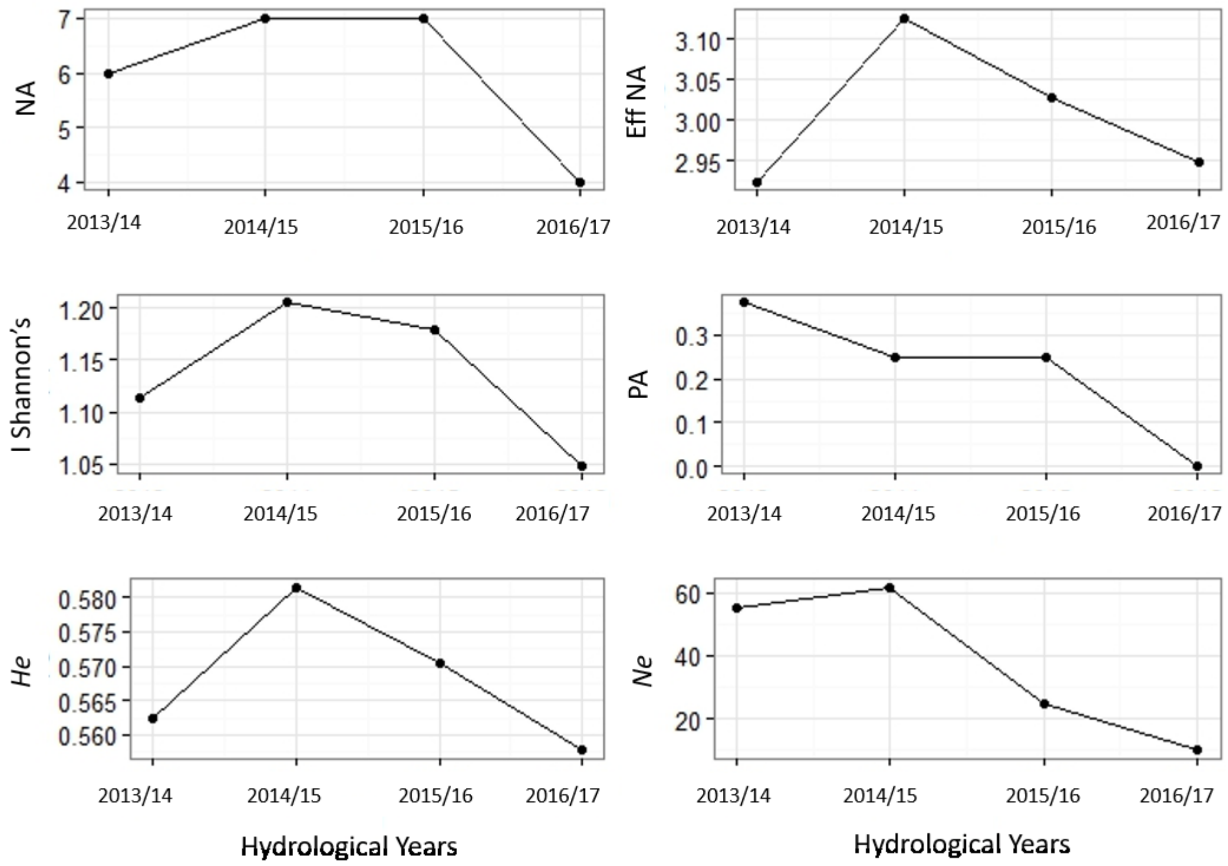

II. Factorial Correspondence Analysis from Genetix, demonstrating the distribution of the genetic patterns of *Prochilodus costatus* individuals sampled from Migratory and Non-Migratory periods.

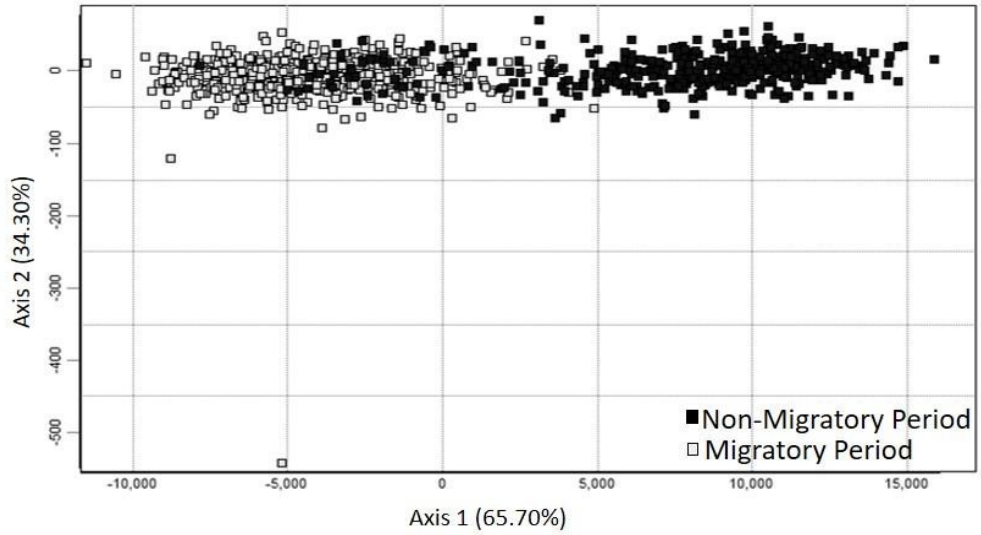

III. Details of microsatellites' identification and genotyping for *P. costatus* populations from upper São Francisco river Basin in Brazil. These information were published in Pimentel et al. (2018; doi: 10.3389/fgene.2018.00073) and were kindly provided to compose this material.

To improve reliability, we trimmed and filtered the obtained reads using the PRINSEQ tool (Schmieder and Edwards, 2011). Bases with Phred scores lower than 30 and/or read lengths shorter than 75 bp were removed. Filtered reads were aligned against a FASTA file containing reference sequences for the eight microsatellite loci using the software Bowtie 2 (Langmead and Salzberg, 2012) with the high sensitivity option. Alignment against a reference region that contains insertions or deletions of nucleotides, such as the microsatellite variants requires careful curation because variations in the edge of the repeat can lead to error in alignment and consequent misidentification of the alleles. One way to increase the confidence and reduce error is to realign the reads taking into account the nucleotide sequence of the edges of the repeat and possible variants within the region. As no information on the variants of the microsatellite region studied here was available in public databases, we identified the possible variants at the edges of the repetition of each of the eight loci. We used the SAMtools package (Li et al., 2009) to detect possible variants in the mapping file (BAM format) of each of the 384 sequenced individuals. As a result, we obtained a Variant Call Format (VCF) file containing all the variants in the regions around the microsatellites repeat motifs. Next, we realigned the reads that mapped to the reference repeating regions using the tools RealignTargetCreator and IndelRealigner from the GATK package (McKenna et al., 2010). These tools perform a local realignment to the regions of the repeat motifs taking into account only high-quality reads that completely cover the repeat region and the variants described for the region (VCF file). Reads that did not match these criteria were removed. A realignment file (BAM format) containing only reads that realigned to each locus (tested twice) was obtained for each individual, thus increasing the confidence in the identification of the alleles. We used the RepeatSeq tool (Highnam et al., 2012), with parameter M 2 (minimum sequencing quality required value) to identify and quantify the alleles from the realignment files. This tool requires a file containing the chromosome coordinates and the repeat region motif sequence. Since P. costatus genomic information was unavailable, we used the information obtained through the microsatellites amplicon sequencing as an independent chromosome. We created an input file containing the name, the starting and ending positions of the repeat sequence in the amplicon, and the sequence of repeat motif for each locus. The information of the chromosomal regions was replaced by the information of each of the amplicons. There is no limit regarding the size or number of amplicons. However, it is important to enter the correct location and base sequence of the repeat region. The RepeatSeq tool uses the coordinate file to search for repeat regions in the realignment files and calculates the repeat length, which determines the alleles. A repeat ATTATTATTATT, for example, would be defined as allele 12. After identification of the repeat motif, the reads that aligned to that region are selected and quantified, according to their number of repeats. The resulting file contains the full read annotation of the reference microsatellite, including the total number of alleles detected, total number of reads, total number of reads per allele, and mapping quality score. To avoid false negatives and to convert the results into the input format required by the software commonly used in population genetics, we developed a Perl script, named GenotypeMicrosat.pl. This script performs a detailed analysis of the RepeatSeq output file. We determined the individual's genotype for each locus using the following filter criteria: (1) maximum of two alleles per individual per locus; (2) at least 10 reads per locus in the entire repeat sequence, including eight bases in the 50 and eight bases in the 30 flanking regions; and (3) at least 20% of reads corresponding to a second allele for an individual to be considered heterozygous in a given locus. Individuals with a second allele coverage of less than 20% were considered homozygous. Following application of these filter criteria, we generated a spreadsheet containing the genotypes of each individual for each of the eight loci. Loci that did not attain the filter requirements were identified as 'NA.' The generated spreadsheet can be easily adapted for other population genetics analysis programs.

VI. Hardy-Weinberg and Null allele's results.

Summary of Chi-Square Tests for Hardy-Weinberg Equilibrium

Pop	Locus	DF	ChiSq	Prob	Signif
PA1	Pcos10	78	1323.000	0.000	***
PA1	Pcos18	120	1690.000	0.000	***
PA1	Pcos22	561	3575.000	0.000	***
PA1	Pcos36	91	1770.042	0.000	***
PA1	Pcos37	231	992.000	0.000	***
PA1	Pcos44	946	4977.340	0.000	***
PA1	Pcos48	231	1976.000	0.000	***
PA1	Pcos49	1378	3524.889	0.000	***
PA2	Pcos10	28	170.674	0.000	***
PA2	Pcos18	91	256.397	0.000	***
PA2	Pcos22	561	1829.451	0.000	***
PA2	Pcos36	120	796.458	0.000	***
PA2	Pcos37	21	50.000	0.000	***
PA2	Pcos44	276	617.491	0.000	***
PA2	Pcos48	120	199.800	0.000	***
PA2	Pcos49	1275	2486.157	0.000	***
SFU	Pcos10	45	244.667	0.000	***
SFU	Pcos18	136	396.917	0.000	***
SFU	Pcos22	528	1693.202	0.000	***
SFU	Pcos36	153	753.025	0.000	***
SFU	Pcos37	253	840.914	0.000	***
SFU	Pcos44	741	1379.834	0.000	***
SFU	Pcos48	171	488.278	0.000	***
SFU	Pcos49	1378	2764.846	0.000	***
SFD	Pcos10	45	646.233	0.000	***
SFD	Pcos18	153	810.199	0.000	***
SFD	Pcos22	703	3924.118	0.000	***
SFD	Pcos36	210	2566.058	0.000	***
SFD	Pcos37	276	1353.427	0.000	***
SFD	Pcos44	946	3064.083	0.000	***
SFD	Pcos48	300	982.784	0.000	***
SFD	Pcos49	1596	6148.042	0.000	***
PAO	Pcos10	28	1320.000	0.000	***
PAO	Pcos18	210	4250.000	0.000	***
PAO	Pcos22	351	5633.138	0.000	***
PAO	Pcos36	78	1936.261	0.000	***
PAO	Pcos37	378	4494.000	0.000	***
PAO	Pcos44	780	6029.521	0.000	***
PAO	Pcos48	231	2475.000	0.000	***
PAO	Pcos49	1128	5537.763	0.000	***

Key: ns=not significant, * P<0.05, ** P<0.01, *** P<0.001

Locus	Pop	Null alleles frequency	Locus	Pop	Null alleles frequency
1	1	0.000	5	1	0.022
1	2	0.020	5	2	0.000
1	3	0.079	5	3	0.000
1	4	0.000	5	4	0.017
1	5	0.087	5	5	0.086
1	6	0.055	5	6	0.022
1	7	0.000	5	7	0.014
1	8	0.000	5	8	0.028
1	9	0.000	5	9	0.001
1	10	0.019	5	10	0.001
1	11	0.050	5	11	0.024
1	12	0.000	5	12	0.000
1	13	0.097	5	13	0.037
1	14	0.030	5	14	0.001
1	15	0.000	5	15	0.018
1	16	0.000	5	16	0.028
2	1	0.068	6	1	0.031
2	2	0.000	6	2	0.021
2	3	0.000	6	3	0.022
2	4	0.049	6	4	0.027
2	5	0.000	6	5	0.015
2	6	0.000	6	6	0.031
2	7	0.037	6	7	0.014
2	8	0.013	6	8	0.025
2	9	0.000	6	9	0.020
2	10	0.000	6	10	0.027
2	11	0.068	6	11	0.031
2	12	0.000	6	12	0.001
2	13	0.000	6	13	0.015
2	14	0.012	6	14	0.015
2	15	0.015	6	15	0.010
2	16	0.000	6	16	0.020
3	1	0.042	7	1	0.022
3	2	0.074	7	2	0.017
3	3	0.094	7	3	0.017
3	4	0.020	7	4	0.023
3	5	0.059	7	5	0.097
3	6	0.007	7	6	0.036
3	7	0.029	7	7	0.018
3	8	0.011	7	8	0.021
3	9	0.021	7	9	0.000
3	10	0.028	7	10	0.016
3	11	0.016	7	11	0.021
3	12	0.010	7	12	0.000
3	13	0.018	7	13	0.023
3	14	0.015	7	14	0.021
3	15	0.093	7	15	0.022
3	16	0.018	7	16	0.015
4	1	0.000	8	1	0.010
4	2	0.018	8	2	0.026
4	3	0.042	8	3	0.021
4	4	0.000	8	4	0.000
4	5	0.010	8	5	0.000
4	6	0.001	8	6	0.000
4	7	0.000	8	7	0.011
4	8	0.000	8	8	0.018
4	9	0.000	8	9	0.016
4	10	0.049	8	10	0.099
4	11	0.000	8	11	0.019
4	12	0.000	8	12	0.024
4	13	0.000	8	13	0.028
4	14	0.013	8	14	0.019
4	15	0.000	8	15	0.016
4	16	0.076	8	16	0.015

V. DAPC script and result of assignment genotype of individuals of P. costatus into k-clusters (k=3) from Figure 1A.

```
>library(adegenet)
>data=read.genepop("data.gen")
>data
>bic=find.clusters(data,max.n.clust=23,choose=FALSE)
Choose the number PCs to retain (>=1): 55
>bic
>bic$stat
>bic$grp
> bic$grp
>dapc(data, bic$grp)
Choose the number of PCs to retain (>=1): 55
Choose the number of discriminants function to retain (>=2): 6
>compoplot(dapc)
```
